# Increased glucocorticoid receptor expression in sepsis is related to heat shock proteins, cytokines, and cortisol and is associated with increased mortality

**DOI:** 10.1186/s40635-017-0123-8

**Published:** 2017-02-21

**Authors:** Konstantinos Vardas, Stavroula Ilia, Amalia Sertedaki, Evangelia Charmandari, Efrossini Briassouli, Dimitris Goukos, Kleovoulos Apostolou, Katerina Psarra, Efthimia Botoula, Stylianos Tsagarakis, Eleni Magira, Christina Routsi, Constantine A. Stratakis, Serafim Nanas, George Briassoulis

**Affiliations:** 1 First Critical Care Department, National and Kapodistrian University of Athens, Athens, Greece; 2 Pediatric Intensive Care Unit, University Hospital, University of Crete, 71500 Heraklion, Crete Greece; 3 Division of Endocrinology, Metabolism and Diabetes, First Department of Pediatrics, ‘Aghia Sophia’ Children’s Hospital and Division of Endocrinology and Metabolism, Biomedical Research Foundation of the Academy of Athens, National and Kapodistrian University of Athens, Athens, Greece; 4 First Department of Internal Medicine-Propaedeutic, National and Kapodistrian University of Athens, Athens, Greece; 5 Immunology-Histocompatibility Department, Evangelismos Hospital, Athens, Greece; 6 Department of Endocrinology-Diabetes, Evangelismos Hospital, Athens, Greece; 7 Section on Endocrinology and Genetics, Eunice Kennedy Shriver National Institute of Child Health and Human Development (NICHD), National Institutes of Health, Bethesda, MD 20892 USA

**Keywords:** Glucocorticoid receptor (GR), Cortisol, Heat shock protein 72, HSP90α, Sepsis, SIRS

## Abstract

**Background:**

The purposes of this study are to examine if the human glucocorticoid receptor (hGR) isoform-α mRNA and hGR protein expressions are deficient in the acute phase of sepsis (S) compared to systemic inflammatory response syndrome (SIRS) and healthy subjects (H) and to evaluate if the hGRα and hGR alterations are associated with cortisol changes and if they are related to (1) extracellular and intracellular heat shock proteins (HSP) 72 and 90α; (2) ACTH, prolactin, and interleukins (ILs); and (3) outcome.

**Methods:**

Patients consecutively admitted to a university hospital intensive care unit (ICU) with S (*n* = 48) or SIRS (*n* = 40) were enrolled in the study. Thirty-five H were also included. Total mRNA was isolated from peripheral blood samples and cDNA was prepared. RT-PCR was performed. Intracellular hGR and HSP expression in monocytes and/or neutrophils was evaluated using four-colour flow cytometry. Serum prolactin, ACTH, and cortisol concentrations were also measured. ELISA was used to evaluate serum ILs and extracellular (e) HSPs (eHSP72, eHSP90α).

**Results:**

hGR protein was higher in S compared to H and SIRS; hGRα mRNA was higher in S compared to H (*p* < 0.05). In sepsis, hGR protein and eHSP72 were higher among non-survivors compared to survivors (*p* < 0.05). The hGR MFI and hGRα mRNA fold changes were significantly related to each other (*r*_s_ = 0.64, *p* < 0.001). Monocyte hGR protein expression was positively correlated with extracellular and intracellular HSPs, cortisol, and ILs and negatively to organ dysfunction (*p* < 0.05). HSPs, hGR, and cortisol were able to discriminate sepsis from SIRS (AUROC > 0.85, *p* < 0.05). In sepsis, monocyte-hGR protein and eHSP72 were strong predictors of mortality (AUROC > 0.95, *p* < 0.04).

**Conclusions:**

Acute-phase sepsis is associated with increased hGR expression and cortisol concentrations, possibly implying no need for exogenous steroids. At this stage, hGR is able to predict sepsis and outcome and is related to stress-activated bio-molecules and organ dysfunction.

## Background

Sepsis is a life-threatening organ dysfunction caused by a dysregulated host response to infection [1]. The activation of the hypothalamic–pituitary–adrenal (HPA) axis results in increased cortisol concentrations driven by increased secretion of ACTH [2]. Also, decreased activity of the cortisol metabolizing enzymes impairs cortisol clearance, enhances hypercortisolism, and suppresses ACTH secretion by feedback inhibition [3].

In humans, glucocorticoids (GCs) exert their effects through binding to their cognate receptor, the glucocorticoid receptor, a ligand-dependent transcription factor [4]. The human glucocorticoid receptor (hGR) is encoded by the *NR3C1* gene, which is located on the long arm of chromosome 5 (5q31.3) and is composed of ten exons. Alternative splicing of exon nine generates two highly homologous receptor isoforms, hGRα, and hGRβ. The residing in the cytoplasm hGRα functions as a ligand-dependent transcription factor [4]. The hGRβ does not bind glucocorticoid agonists and exerts a dominant negative effect on the transcriptional activity of hGRα [5].

**Fig. 1:**
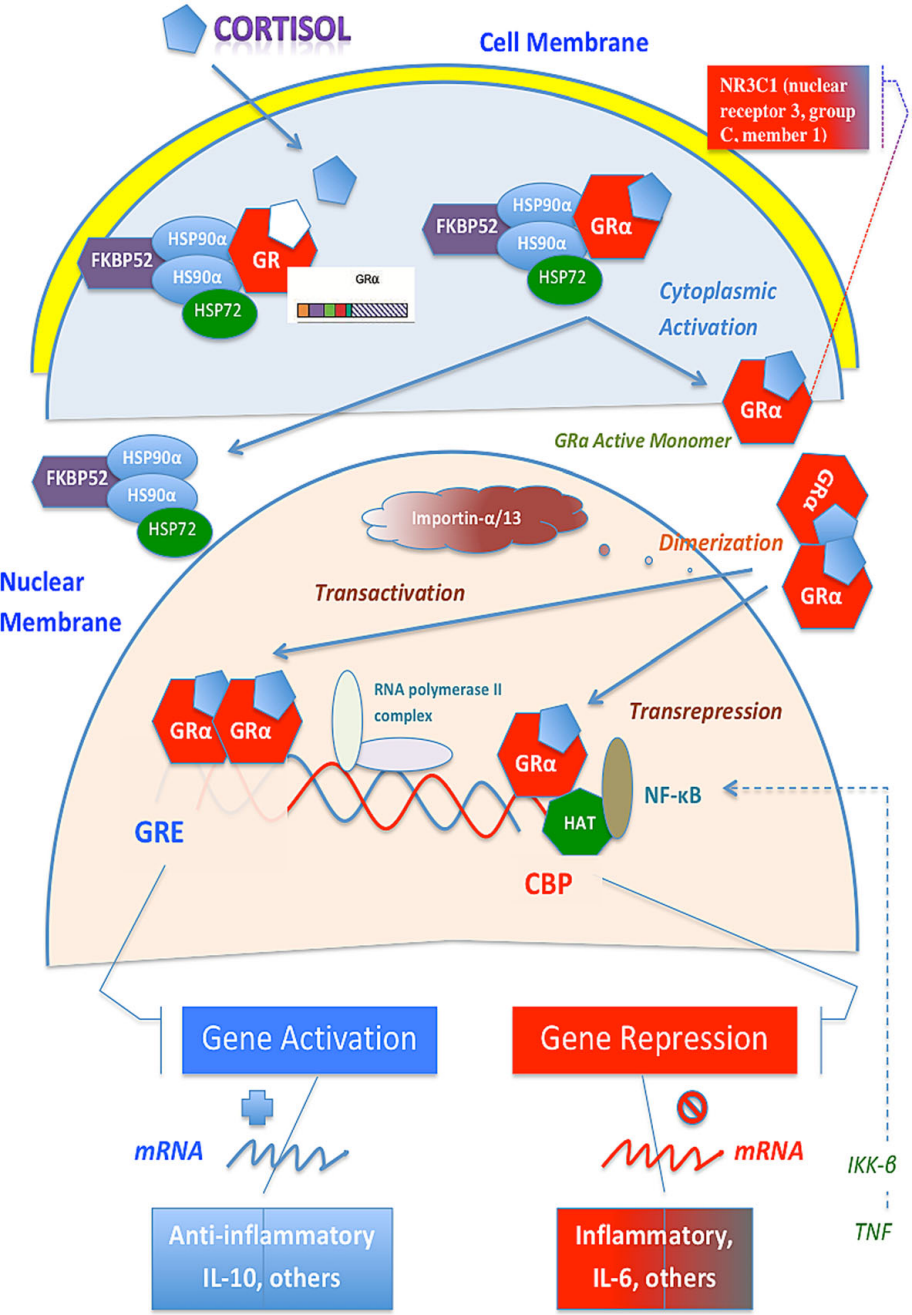
Glucocorticoids (GC) diffuse across the cell membrane and bind to human glucocorticoid receptor (hGR) in the cytoplasm. In a heat shock protein (HSP) heterocomplex, hGRα is activated (upon ligand binding), is released from HSP72 and HSP90α, and rapidly translocates into the nucleus, where the transcription of target genes is initiated. Through transactivation, binding of two hGRα molecules together as a homodimer to glucocorticosteroid response elements (GRE) in the promoter region of steroid-sensitive genes leads to the transcription of genes encoding anti-inflammatory mediators (i.e., IL-10) and the inhibition of nuclear factor-κB (NF-κB). Through transrepression, the hGRα–GC complex interacts with the activated by NF-κB and other pro-inflammatory transcription factors with intrinsic histone acetyltransferase (HAT) activity switching off multiple activated inflammatory genes (i.e., IL-6). *CBP cAMP* response element binding protein, *IKKβ* inhibitor of I-κB kinase-β

In the absence of ligand, the hGRα resides mostly in the cytoplasm of cells as part of a hetero-oligomeric complex, which contains chaperone heat shock proteins (HSPs) 90 and 70. HSP90 regulates ligand binding, as well as cytoplasmic retention of hGRα by exposing the ligand-binding site and masking the two nuclear localization sequences [4]. HSP90α and HSP72 work together as the only two essential components of the five-protein system for allowing hGRα to bind the incoming steroid hormone by enhancing its affinity for the ligand [6]. Upon ligand binding and phosphorylation, the hGRα dissociates from HSPs and translocates into the nucleus. Nuclear hGRα binds to glucocorticoid response elements (GREs) of target genes and upregulates or downregulates their expression, depending on GRE sequence and promoter context [4] (Fig. 1).

GCs downregulate the expression of their receptor through transcriptional, posttranscriptional, and posttranslational mechanisms [7]. Thus, treatment with GCs activates a negative feedback loop by inducing a specific critical suppressor of *NR3C1* gene, the microRNA-124, thereby impeding the hGRα upregulation and shifting the hGRα:hGRβ ratio toward hGRβ. Such a shift limits or negatively impacts the GC anti-inflammatory effects by aggravating the GC resistance [8]. Septic serum induces the expression of not only hGRα but also of hGRβ in both T and B cells in culture, supporting a possible GC resistance of specific cell subpopulations in sepsis [9]. In pediatric sepsis, however, hGRβ mRNA levels did not change significantly during sepsis, verifying inconsistency with results of hGRβ [10]. By investigating hGRα mRNA expression rather than protein levels [8], these studies produced inconsistent results about the hGRα expression and function in critical illness.

To the best of our knowledge, the assessment of both protein and mRNA hGRα expression in sepsis and systemic inflammatory response syndrome (SIRS) in relation to their hetero-oligomeric HSPs parts, and the stress-induced inflammatory response has not been investigated previously. In this study, we assessed the hGR protein expression in monocytes and the hGRα mRNA in whole blood in intensive care unit (ICU) patients with early-onset sepsis (S) compared to SIRS or healthy control subjects (H). We examined the hypothesis that the hormonal-innate immune stress-responding pairs of hGR-cortisol and hGR-HSPs are adequately expressed in the acute phase of sepsis. We also examined the hypothesis that the hGR mRNA fold change and protein expression alterations are associated with simultaneous cortisol, extracellular or intracellular HSP72 or HSP90α changes, or with stress-activated bio-molecules and organ dysfunction. Finally, we evaluated the ability of the hGR alterations to predict sepsis or outcome.

## Methods

### Patients

The study was approved by the institutional review board of Evangelismos Hospital and was performed during a 24-month period between September 2013 and September 2015. Written informed consent was obtained from the relatives of patients admitted to the ICU. Patients aged 18–75 years consecutively admitted with early-onset (<24 h) sepsis/septic shock or SIRS were eligible for enrollment. The sepsis group (S) included patients (*n* = 48) with an identified source of infection and Sequential Organ Failure Assessment (SOFA) [11] score >2, according to the updated Sepsis-3 definition [12]. Of those, 59.5% fulfilled the septic shock criteria (need for inotropic support to maintain MAP > 65 mmHg and lactate levels >2 mmol/L). The non-infectious SIRS group included trauma patients (*n* = 40) who met at least two of the four conventional criteria for SIRS [13] and represented the first control group (ICU control). The H group included healthy volunteers (*n* = 35), representing the second control group (healthy individuals). Exclusion criteria were (a) malignancy, (b) autoimmune diseases, (c) prior use of corticoids, (d) immune deficiency disorders, and (e) late sepsis or SIRS > 48 h after admission. Acute Physiology and Chronic Evaluation-II (APACHE II) [14], multiple organ dysfunction (MODS) [15], Simplified Acute Physiology Score-III (SAPS III) [16], and SOFA scores were recorded on admission. Blood samples were obtained at 8 am because of a peripheral CLOCK-mediated circadian acetylation of the human GR counter-regulating the actions of diurnally fluctuating cortisol [17]. Blood gas values and clinical laboratory data were obtained from laboratory records.

### Laboratory assays

#### RNA isolation, reverse transcription and RT-PCR for GRα (NR3C1)

Total RNA was isolated from peripheral blood employing the TRIzol Reagent (Ambion by Life Technologies) according to the manufacturer’s instructions. RNA (1 μg) was DNase treated using RQ1 RNase-free DNase (Promega) and reverse transcription was carried employing the Transcriptor First Strand cDNA Synthesis Kit (Roche Applied Science). Duplicate samples of the cDNA underwent employing primers and TaqMan probes designed by TIB MOLBIOL and the LightCycler 480 Probes Master Kit (Roche) on a LightCycler 480 System (Roche). The reference gene used in this study was the RPLP0. Repeated trials for hGRβ detection in samples of all groups were mostly unsuccessful, so that only hGRα mRNA was measured. Standard curves were constructed using triplicate samples of a standard RNA sample for hGRα and RPLP0. The sequence of the primers and probes of the target and the reference gene were:

- hNR3C1a S 5’-TATgCATgAAgTggTTgAAAATCTCC-3’(exon 9)
- hNR3C1a A 5’-ggTATCTgATTggTgATgATTTCAgC-3’ (exon 9)
- hNR3C1 FAM-CATCTCggggAATTCAATACTCATggTCTT-BBQ (exon 9)
- RPLP0-F 5’-CTCTGGAGAAACTGCTGCCTCATA-3’, (exon 4)
- RPLP0-R 5’-GACTTCACATGGGGCAATGG-3’, (exon 5)
- RPLP0 FAM-AGGACCTCACTGAGATCAGGGACATGT-BBQ (exon 4-5)

Data analysis was performed using the Advanced Relative Quantification software of the LightCycler 480.

#### Flow cytometry for human GR, HSP72, and HSP90α

The monocytes and neutrophils protein expression was evaluated by flow cytometry. Ethylenediaminetetraacetic acid (EDTA)-anticoagulated blood (100 μL) was used for flow cytometric analysis of fresh peripheral blood monocyte (m) and neutrophil (n) HSP72 and HSP90α, and monocytes hGRα protein expressed as mean fluorescence intensity (MFI) were determined after staining surface antigens CD33 and CD45 with 5 μL monoclonal antibodies CD33-PE/Cy5 and CD45 PE/Cy7 (BioLegend, San Diego, USA) followed by HSP72, HSP90α, and hGR intracellular staining with 5 μL HSP72-FITC, 5 μL HSP90α-PE, or 10 μL anti-hGR-FITC monoclonal antibodies, respectively (Enzo Life Sciences, Ann Arbor, MI, USA). Anti-hGRα antibodies conjugated to any fluorescence were not commercially available. Additionally, although hGR antibody detects all protein isoforms, hGRα is represented in almost 99% of hGR protein [10]. Non-detection of hGRβ protein with flow cytometry would also be expected when it couldn’t be detected on RNA level. Isotype controls were used to check if blocking was needed. Assays were performed according to the manufacturer’s instructions using Flow Cytometer FC-500 (Beckman Coulter, Miami, FL, USA).

#### ACTH cortisol and prolactin

During the first 48 h after admission to ICU, blood samples for determination of ACTH cortisol and prolactin were collected at 08:00 h. Plasma ACTH was measured using the Immulite 2000 Immunoassay Analyzer (Siemens Healthcare Diagnostics, Tarrytown, NY, USA), while serum cortisol and prolactin concentrations were determined using the ADVIA Centaur Immunoassay Analyzer (Siemens Healthcare Diagnostics, Tarrytown, NY, USA).

#### Cytokines and extracellular heat shock proteins

Serum cytokine concentrations of IL-6, IL-10, IL-17, and interferon (IFN)-γ were measured by ELISA according to the instructions of the manufacturer. Extracellular plasma levels of HSPs (eHSP72 and eHSP90α) were analyzed by ELISA assay according to the manufacturers’ instructions (Invitrogen Carlsbad, CA, USA and Enzo Life Sciences, Ann Arbor, MI, USA, respectively). Inter- and intra-assay confidence intervals (CI) for each analyte were: IL-6:6.2, 7.8; IL-10:3.25, 2.75 IL-17:3.7 and in process, IFN-γ:3.5, 7.3, HSP72:7.1, 15.2, HSP90α < 10. Sensitivities of assays: IL-6 < 2 pg/mL, IL-10 < 1 pg/mL, IL-17 = 2 pg/mL, IFN-γ = 0.03 IU/mL, HSP72 = 90 pg/mL, and HSP90α = 50 pg/mL.

### Statistical analysis

The one-sample Kolmogorov–Smirnoff test was used to determine the data distribution from measured variables. Data are expressed as median values with range for continuous parameters and as frequencies for categorical variables. The Kruskal–Wallis independent samples test was used to perform comparisons among groups, as appropriate. We ran multiple comparison analyses using post hoc Dunn’s pairwise tests with Bonferroni corrections for the Kruskal–Wallis *H* test; the *x* ^2^ test statistic (*H* ^2^) was adopted to calculate the effect size (*w* ^2^ *= H* ^2^ */*(*n −* 1)) to avoid the inflation of overall type I error. Between-group comparisons were conducted using the *χ* ^2^ test for categorical parameters and Spearman’s rank correlation coefficient for correlation between two continuous variables. The effect size for *x* ^*2*^ test was measured using the Cramer’s *V* (*V = √x* ^2^ */n*.df) goodness of fit*.* To evaluate predictive values, we calculated the areas under the receiver operating characteristic curves (AUROC) for variables significantly differing between S and SIRS groups or between survival and non-survival sub-cohorts for the sepsis group. For the predictive values of sepsis or mortality we used biomarkers significantly related to them in bivariate correlations, as appropriate. We consider an AUROC > 0.80 as clinically relevant whereas the optimum cutoff value was calculated by the highest sensitivity and specificity combined (Youden index approach). The positive and negative predictive values were also calculated. A two-sided significance level of 0.05 was used for statistical inference. All statistical analyses were performed using the IBM SPSS Statistics (version 22.0; Chicago, IL).

## Results

### Group differences

Monocytes hGR protein expression was higher in S compared to H and SIRS (Kruskal–Wallis Test, pairwise comparisons); hGRα mRNA was higher in S compared to H (Table 1). Groups also differed regarding severity of illness, hormonal, inflammatory, intracellular, and extracellular innate immune response. The proportions of variability (effect size) in the ranked dependent variables accounted for by grouping variables were >0.20 (medium per Cohen), indicating a fairly strong relationship between sepsis and the expression levels of hGR protein, cortisol, extracellular HSP72 and 90α, SAPS-III, IL-6, procalcitonin (PCT), age, sex, and mortality (Table 1).

**Table 1:** Baseline characteristics at study enrollment and group differences

	Healthy subjects (*n* = 35)	SIRS (*n* = 40)	Sepsis (*n* = 48)^d^	Statistic value *x* ^2^ or *H* ^2^	Effect size (*w* ^2^ or *V*)**	*P* value***
Age (years)^e^	35 (28–50)^a^	41 (32–53)^c^	58 (44–69)^a, c^	34	0.29	<0.001
Gender (male/female)	19/16	31/9	28/20	5.2	0.21	0.07
Mortality in ICU (died/survived (%))	0/35	5/35 (12.5%)	21/27 (43.8%)	10.2	0.34	0.001
Mortality in hospital (died/survived (%))	0/35	6/34 (15.8%)	29/19 (60.4%)	17.5	0.45	<0.001
LOS (days)	NA	15.5 (7–28.5)	18.5 (9.2–36.8)	2	0.03	0.16
APACHE II	NA	15 (10–18)^c^	21 (16–27)^c^	15	0.19	<0.001
SOFA	NA	9 (7–11)^c^	11 (9–13)^c^	11	0.14	0.001
SAPS III	NA	41 (43–58)^c^	41 (61–78)^c^	32	0.44	<0.001
CRP (mg/dl)	NA	7.1 (1.05–15)^c^	21 (8.8–27)^c^	10.5	0.15	0.001
PCT (ng/ml)	NA	0.9 (0.5–1.8)^c^	5.2 (1–34)^c^	7	0.23	0.008
Lactate	NA	2.4 (1.4–4.2)	2.8 (1.3–5)	0.23	0.00	0.63
Glucose (mg/dl)	NA	154 (128–189)^c^	219 (145–299)^c^	8.6	0.12	0.003
Albumin (g/dl)	NA	3 (2.3–3.4)^c^	2.5 (2.1–2.9)^c^	6.3	0.09	0.012
WBC (K/μl)	NA	11.4 (7.3–15.5)	10.9 (2.6–19.9)	0.12	0.00	0.91
hGRα mRNA (fold change)	0.50 (0.12–0.9)^a^	0.64 (0.32–0.77)	0.74 (0.52–1.23)^a^	8.3	0.08	0.016
hGR (MFI)	0.95 (0.76–1.9)^a^	1.1 (0.43–2.5)^c^	3.3 (1.6–4.6)^a, c^	9.6	0.28	0.008
Prolactin (ng/ml)	7.5 (5–10)^a b^	18.3 (11–25)^b^	15.4 (9–32)^a^	20.4	0.17	<0.001
Cortisol (μg/dl)	12.3 (9–15)^a^	16.2 (11–23)^c^	32.8 (21–67)^a, c^	38.6	0.33	<0.001
ACTH (pg/ml)	17.5 (13–33)^b^	10 (6–19)^b, c^	18.6 (10–38)^c^	10.4	0.09	–0.005
eHSP90α (ng/ml)	10.5 (1.2–23)^a, b, c^	32 (22–57)^b, c^	71 (31–124)^a, c^	39.5	0.33	<0.001
eHSP72 (ng/ml)	0.26 (0.14–0.41)^a^	0.29 (0.19–0.49)^c^	0.95 (0.41–2.5)^a, c^	32	0.29	<0.001
mHSP90α (MFI)	24 (12–82)	36 (10.5–64)	33 (7.6–56)	0.07	0.00	0.96
mHSP72 (MFI)	19.6 (13–32)	22.8 (11–52)	18 (11.7–30.7)	0.84	0.01	0.65
nHSP90α (MFI)	11 (5.4–25)	17 (4.8–25)	18.3 (6.8–38)	3.5	0.03	0.17
nHSP72 (MFI)	77 (44–110)^a^	75 (28–154)^c^	40 (25–65)^a, c^	13.7	0.12	0.001
IL-6 (pg/ml)	1.4 (0.98–2.5)^a, b^	77 (21–213)^b^	203 (60–539)^a^	72	0.60	<0.001
IL-10 (ng/ml)	0.01 (0.1–9.5) ^a^	4.9 (0.1–11)^c^	48 (0.1–58)^a, c^	15.6	0.13	<0.001
IL-17 (ng/ml)	0.2 (0.2–33)	0.2 (0.2–6.4)	0.2 (0.2–7.8)	0.05	0.00	0.98
IFN-γ (pg/ml)	6.2 (0.44–9.8)	4.7 (0.13–7.8)^c^	9.7 (4–17)^c^	12	0.10	0.03

*APACHE* Acute Physiology and Chronic Evaluation, *CRP* C-reactive protein, *hGRα* human glucocorticoid receptor α-isoform, *eHSP* extracellular heat shock protein, *nHSP* neutrophil-HSP, *mHSP* monocyte-HSP, *IFN-γ* interferon gamma, *IL* interleukin, *MFI* mean fluorescence intensity, *PCT* procalcitonin, *SAPS III* Simplified Acute Physiology Score, *SIRS* systematic inflammatory response syndrome, *SOFA* Sequential Organ Failure Assessment, *WBC* white blood cells

**Per Cohen for df = 2, the effect size should be viewed as small 0.07, medium 0.21, and large 0.35

***Asymptomatic significance (two-sided test) using the independent samples Kruskal–Wallis test; a *p* value <0.05 was considered statistically significant

^a–c^Dunn-Bonferroni multiple comparisons (all pairwise): ^a^ *p* < 0.05 between healthy subjects and S groups, ^b^ *p* < 0.05 between healthy subjects and SIRS groups, ^c^ *p* < 0.05 between S and SIRS groups

^d^Septic shock, *n* = 25 (60%)

^e^Data are expressed as median (IQR, interquartile range)

None of the patients had received steroids the last month before admission (exclusion criteria). Fifteen S patients had been started on a stress-dose of hydrocortisone before blood sampling because of catecholamine resistant shock (31%). Neither hGR mRNA nor MFI differed between the steroid and non-steroid group. Although HSP90α did not differ between groups, eHSP72 was higher and nHSP72 was lower in the group receiving hydrocortisone (stress dose). Prolactin concentrations were higher but ACTH were lower in females compared to males (*p* < 0.001). Cortisol, hGR, hGRα mRNA, HSPs, and ILs did not differ between males and females.

### Monocytes hGR bivariate correlations

**Fig. 2:**
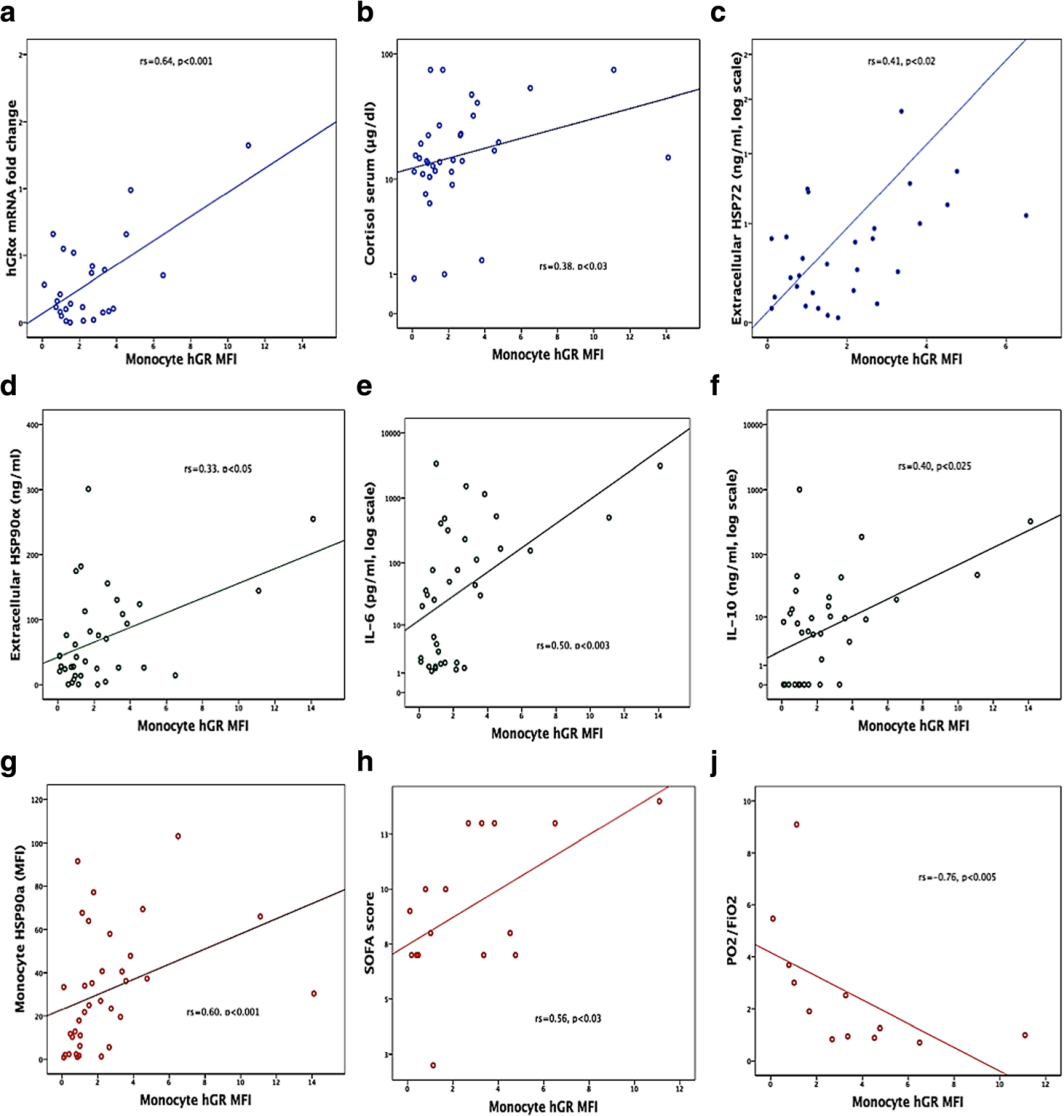
Bivariate correlations of monocyte hGR protein (MFI) with **a** hGRα mRNA (*r*_s_ = 0.64, *p* < 0.001); **b** cortisol (*r*_s_ = 0.38, *p* < 0.03); **c** eHSP72 (*r*_s_ = 0.41, *p* < 0.02); **d** eHSP90α (*r*_s_ = 0.33, *p* < 0.05); **e** IL-6 (*r*_s_ = 0.50, *p* < 0.003); **f** IL-10 (*r*_s_ = 0.40, *p* < 0.025); **g** mHSP90α (*r*_s_ = 0.60, *p* < 0.001); **h** SOFA score (*r*_s_ = 0.56, *p* < 0.03); **j** PO_2_/FiO_2_ (*r*_s_ = −0.76, *p* < 0.005) (Spearman’s rank correlation tests). *hGRα* human glucocorticoid receptor isoform-α, *HSP* heat shock protein, *IL* interleukin, *e* extracellular *m* monocyte, *SOFA* Sequential Organ Failure Assessment

The hGR MFI and hGRα mRNA fold change were significantly related to each other (*r*_s_ = 0.64, *p* < 0.001), even when the hGR protein expression was corrected by the percentages of WBC monocytes (*r*_s_ = 0.59, *p* < 0.01). The expression of hGR protein in monocytes was related to eHSP90α (*r*_s_ = 0.33, *p* < 0.05), eHSP72 (*r*_s_ = 0.41, *p* < 0.02), mHSP90α (*r*_s_ = 0.60, *p* < 0.001), nHSP90α (*r*_s_ = 0.56, *p* < 0.001), mHSP72 (*r*_s_ = 0.41, *p* < 0.02), cortisol (*r*_s_ = 0.38, *p* < 0.03), IL-6 (*r*_s_ = 0.50, *p* < 0.003), and IL-10 (*r*_s_ = 0.40, *p* < 0.025) (Fig. 2a–j). hGR MFI was also negatively related to PO_2_ (*r*_*s*_ = −0.72, *p* < 0.02), PO_2_/FiO_2_ (*r*_s_ = −0.76, *p* < 0.005), and albumin (*r*_s_ = −0.77, *p* < 0.01) and positively to MODS (*r*_s_ = 0.61, *p* < 0.01) and SOFA (*r*_s_ = 0.56, *p* < 0.03). hGRα mRNA was related to eHSP72 (*r*_s_ = 0.34, *p* < 0.02), ACTH (*r*_s_ = 0.36, *p* < 0.005), IFN-γ (*r*_s_ = 0.42, *p* = 0.001), C-reactive protein (CRP) (*r*_s_ = 0.25, *p* < 0.05), and WBC (*r*_s_ = 0.38, *p* < 0.002). No significant associations were found between hGRα and APACHE II, SAPS III, temperature, PCT, IL-17, lactate, LDH, or glucose.

### Mortality

**Fig. 3:**
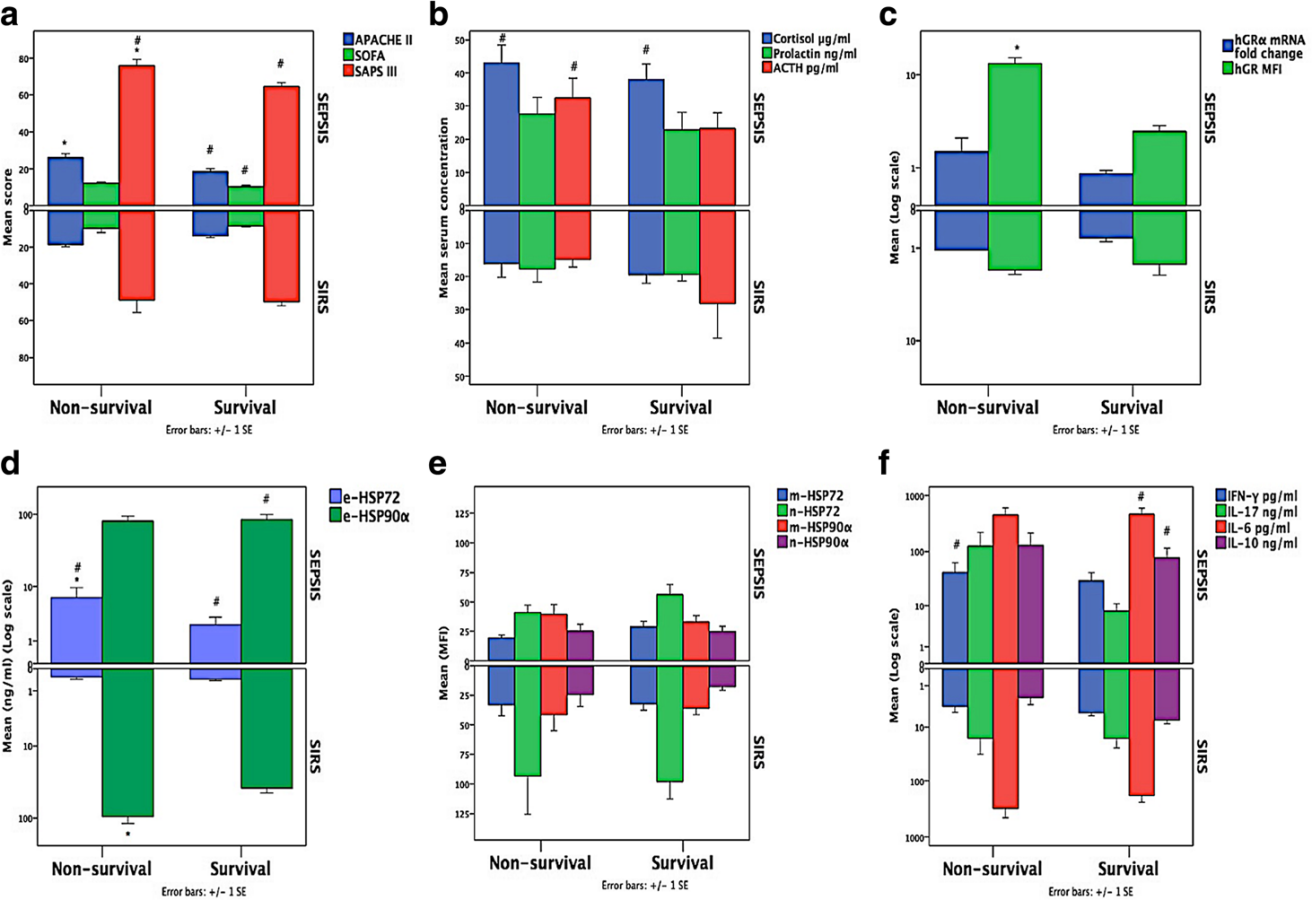
Comparisons between survivors and non-survivors in the sepsis and SIRS subcohorts, regarding **a** severity of illness, **b** hormonal, **c** glucocorticoid receptor, **d** extracellular and **e** intracellular innate immune, and **f** inflammatory acute stress response. The *p* value was calculated using the Mann-Whitney *U* test and a *p* value <0.05 (two sided) was considered statistically significant. Sepsis and/or mortality was associated with or had a trend for increased severity of illness, expression of extracellular HSP90α and HSP72, cytokines, cortisol, and GR and a trend for lower intracellular HSP72. *Asterisk* indicates *p* < 0.05 between non-survivors and survivors in each diagnostic group; *number sign* indicates *p* < 0.05 between sepsis and SIRS in each outcome group. *SIRS* systemic inflammatory response syndrome, *APACHE II* Acute Physiology and Chronic Evaluation, *SOFA* Sequential Organ Failure Assessment, *SAPS III* Simplified Acute Physiology Score III, *GR* glucocorticoid receptor, *HSP* heat shock protein, *IL* interleukin, *IFN-γ* interferon gamma, *MFI* mean fluorescence intensity

More patients with sepsis died compared to those with SIRS (43.8 vs. 12.5%, *p* = 0.001) (Table 1). Within the sepsis group, more patients with septic shock showed a trend to die compared to those without (56 vs. 29%, *p* = 0.08). In sepsis group, APACHE II (*p* < 0.001), SAPS III (*p* < 0.02), hGR protein expression in monocytes (*p* < 0.04), and eHSP72 (*p* < 0.05) were higher among non-survivors compared to survivors. In SIRS, eHSP90α levels (*p* < 0.02) were higher among non-survivors (Fig. 3a–f).

Regarding survivors, when septic and SIRS patients were compared, APACHE II (*p* < 0.03), SOFA (*p* < 0.03), SAPS III (*p* < 0.001), cortisol (*p* = 0.001), eHSP72 (*p* < 0.001), eHSP90α (*p* < 0.02), IL-6 (*p* = 0.005), and IL-10 (*p* < 0.01) were higher among septic compared to SIRS survivors. Regarding non-survivors, SAPS III (*p* < 0.01), cortisol (*p* < 0.02), ACTH (*p* < 0.04), eHSP72 (*p* < 0.05), and IFN-γ (*p* < 0.02) were higher among septic compared to SIRS patients (Fig. 3a–f).

Intracellular mHSP72 and nHSP72 were non-significantly decreased in sepsis compared to SIRS, especially among non-survivors. In the septic shock subcohort nHSP72 and nHSP90α were more repressed compared to non-septic shock (*p* < 0.05), the hGRα mRNA showing a similar trend.

### hGR predictive values

**Fig. 4:**
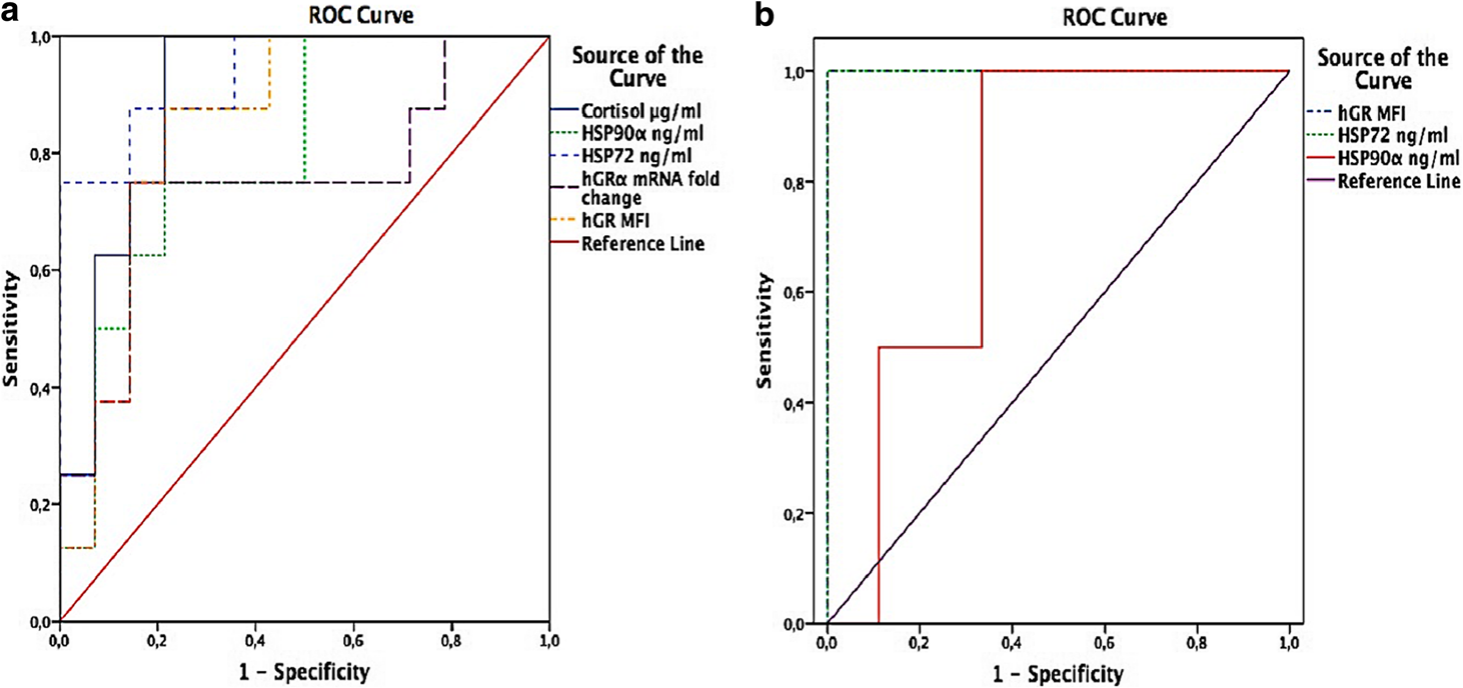
Area under the receiver operating characteristic curve (AUROC) for **a** discriminating sepsis in ICU patients: eHSP72, cortisol, and hGR MFI best discriminated sepsis from SIRS as indicated by an AUROC > 0.85; **b** AUROC for predicting mortality among patients with sepsis (Sepsis-3 definition): eHSP72 and hGR MFI were strong predictors of mortality, achieving an AUROC > 0.95. *hGRα* human glucocorticoid receptor isoform-α*, HSP* heat shock protein, *MFI* mean fluorescence intensity

We generated ROC curves for hGR, stress-proteins (HSPs) expressing innate immunity, and cortisol with the best *p* values for discriminating sepsis among ICU patients (Fig. 4a) or predicting mortality in the subpopulation of Sepsis-3 patients (Fig. 4b). Extracellular HSP72, cortisol, and hGR protein expression in monocyte best-discriminated sepsis from SIRS as indicated by an AUROC > 0.85 with cutoff values and positive and negative predictive values presented in Table 2. In the sepsis group only, monocyte-hGR protein expression and eHSP72 were strong predictors of mortality, achieving an AUROC > 0.95 with cutoff values and positive and negative predictive values presented in Table 3.

**Table 2:** Receiver operating characteristic curve analysis to determine the optimum cutoff values and the positive and negative predictive values of hGR, cortisol, and HSPs for the prediction of sepsis in ICU patients

	Parameters							
Biomarkers	AUROC (95% CI)	*P* value	Sensitivity	Specificity	*J*	Cutoff	PPV (%)	NPV (%)
eHSP72	0.94 (0.84–1.0)	0.001	0.88	0.79	0.66	0.40	73	68
Cortisol	0.90 (0.77–1.0)	0.002	1.00	0.79	0.79	15.7	66	70
hGR MFI	0.85 (0.69–1.0)	0.008	0.88	0.79	0.66	2.66	73	49
eHSP90α	0.80 (0.62–0.99)	0.02	0.75	0.79	0.54	66.0	72	56
hGRα mRNA	0.75 (0.52–0.98)	0.05	0.75	0.86	0.61	0.38	66	67

*Abbreviations*: *AUROC* area under the receiver operating characteristic curve, *CI* confidence interval, *J* Youden’s index, *PPV* positive predictive value, *NPV* negative predictive value, *eHSP* extracellular heat shock protein, *hGR* human glucocorticoid receptor, *MFI* mean fluorescent intensity

**Table 3:** Receiver operating characteristic curve analysis to determine the optimum cutoff values and the positive and negative predictive values of hGR, cortisol, and HSPs for the prediction of mortality in ICU patients with Sepsis-3

	Parameters							
Biomarkers	AUROC (95% CI)	*P* value	Sensitivity	Specificity	*J*	Cutoff	PPV (%)	NPV (%)
hGR MFI	1.00 (1.00–1.0)0	0.034	1.00	0.89	0.89	4.64	66	58
eHSP72	1.00 (1.00–1.00)	0.034	1.00	0.89	0.89	2.1	53	61
eHSP90α	0.79 (0.49–1.00)	0. 23	1.00	0.67	0.67	137.6	44	56

*Abbreviations*: *AUROC* area under the receiver operating characteristic curve, *CI* confidence interval, *J* Youden’s index, *PPV* positive predictive value, *NPV* negative predictive value, *eHSP* extracellular heat shock protein, *hGR* human glucocorticoid receptor, *MFI* mean fluorescent intensity

## Discussion

In this study, we showed that early expression of hGRα mRNA or hGR protein expression in monocytes is significantly higher in patients with sepsis compared to healthy controls and/or patients with SIRS. Furthermore, we verified the hypothesis that the hormonal-innate immune stress-responding pairs of hGR-cortisol and hGR-HSPs are adequately expressed in the early phase of sepsis. We also showed that the hGR mRNA fold change and protein expression alterations are associated with simultaneous cortisol and extracellular or intracellular HSP72 or HSP90α changes. It is possible that hGR is induced in monocytes, in association with the HSPs, to counter-balance the acute inflammatory response early in sepsis. We finally showed that in the acute phase of stress, hGR is able to predict sepsis and outcome and is related with stress-activated bio-molecules and organ dysfunction.

Our finding of upregulation of monocyte hGR expression could represent an adaptive response aiming at dampening the aggressive inflammation in early sepsis. This is in agreement with experimental studies showing increased hGR protein expression and binding capacity in circulating lymphocytes, monocytes, and splenocytes in murine endotoxic shock [18]. Earlier studies had shown lower hGRα mRNA trends in sepsis either in adult T lymphocytes [8] or in neutrophils from children [10] and decreased ^3^H-dexamethasone binding [19]. Importantly, total and cytoplasmic, but not nuclear, hGR protein levels were lower in PBMCs from small groups (*n* = 6–7) of children with sepsis or trauma compared to controls [20]. Earlier research was only done on mRNA, which in our study was shown to produce weaker response compared to protein. Downregulated hGRα in the liver, lung, and spleen but upregulated hGRα mRNA and binding activity have been reported in muscle in endotoxemic rats [21]. Inconsistency in selecting cell subpopulations or methods with different sensitivity in expressing or measuring hGRα might have produced such opposing results.

Although the anti-inflammatory GC actions have been suggested to be more effective in males than in females [22], we did not find any sex-specific influence on hGRα mRNA and hGR-protein expressions, or on serum cortisol, HSP, and ILs concentrations. Similar to our results, others have also observed that there is no correlation between hGRα mRNA levels and admission severity of illness [10]. By generating ROC curves for stress proteins and chaperones, we showed that hGR protein expression in monocytes, stress-proteins (HSPs) expressing innate immunity, and cortisol could discriminate sepsis among ICU patients with acceptable positive and negative predictive values. Acknowledging the limitation of the small sepsis-3 sample, high levels of hGR and eHSP72 were also able to predict mortality among septic patients. These chaperokines (HSPs), expressing innate-immunity, and targeted hormone-response receptors (hGR), seem to represent more sensitive biomarkers in predicting future events than simultaneously calculated or defined severity of illness scores and shock.

hGRα mRNA is produced much more slowly compared to corresponding hGR protein expression, translated at the same time [23]. This is because of several time-consuming steps at RNA processing not required for protein translation. Our finding, showing a good correlation of hGR protein in monocytes with hGRα mRNA, is consistent with reports in other human cells and tissues showing that hGR protein in most cells matches up well with hGRα mRNA levels [24]. Additionally, elevated hGRα mRNA levels correlate with the number of hormone binding sites in septic rats [25] and human lymphocytes [26], leading to increased hGRα binding activity and GC sensitivity [27]. Stimulation of T-cells upregulated the hGRα, possibly rendering T-cells more sensitive to GC, although such a T-cell response was hindered by hydrocortisone [8]. Such a dynamic impact on the expression and function of hGRα might possibly explain why GC are beneficial only when administered early in sepsis [28]. Representing a tissue-specific adaptation, neutrophil hGRα-binding capacity was decreased in murine sepsis [18] and neutrophil hGRα mRNA was decreased in septic children, leading to increased cortisol resistance of neutrophils, gradually normalized after recovery [10]. This is of particular importance since the prone to apoptosis during sepsis lymphocytes present opposing hGRα mRNA regulation from that of the prone to proliferation neutrophils. By adopting a whole-blood mRNA approach, we were able to provide a more comprehensive picture without missing hGR expression signatures from subpopulations that would potentially have been excluded from the experimental approach [29].

We showed for the first time that hGR protein and/or hGRα mRNA is associated with the cell chaperokines expressing innate immunity, extracellular and intracellular HSP72, and HSP90α, besides HPA, IL-6, IL-10, and IFN-γ. Expanding results of previous studies, showing an inverse hGR correlation to noradrenaline and lactate [28], we found that the hGR was also related with CRP, WBC, low PO_2_/FiO2, MODS, and SOFA scores. Although a negative correlation between hyperthermia and the number of receptors has been previously suggested [30], we could not find any relation between hGRα and temperature, lactate, glucose, LDH, IL-17, or PCT. In vitro, only heat shock induced mHSP72 and HSP72 mRNA but not lipopolysaccharide [31]. Our findings are in agreement with those of a recent study showing that in sepsis extracellular HSP72 and HSP90α are increased, while intracellular HSPs are repressed [32]. These data probably imply a key role for the HSP72 and HSP90α early in sepsis in converting the hGRα to the steroid-binding state [6]. The hGRα-cortisol or ACTH relations and the HSP90α time-regulation by the pattern of the pulsatile hormones, further support such a possibility [33].

The increased prolactin concentrations in our septic patients, have been previously shown to inhibit cellular immune functions in septic mice, decreasing survival [34], and to play a role in the acute stress response in sepsis [32]. Generation of cardiotoxic subfragments of prolactin caused by oxidative stress has been implicated in peripartum cardiomyopathy in women [35]. Although pharmacological blockade of prolactin might offer an innovative therapeutic intervention, the exact role of prolactin and its relation to hGRα in sepsis have still to be delineated.

The antibody and methodology used in our study allowed us to measure total hGR levels without distinction of isoforms, which constitutes a limitation of the current study. It is not possible to speculate if the higher hGR protein levels represent proportionally higher hGRβ levels, although we could not detect hGRβ on RNA level. The correlation of hGR protein with hGRα mRNA suggests that hGR is mainly representing hGRα, reportedly expressed in extremely higher concentrations than hGRβ [10]. Weak correlations of hGR with some inflammatory, hormonal, or organ failure indices, although significant, should be interpreted with caution, probably attributed to the small sample numbers. Limitations of this study are also the lack of longitudinal data and the weakness of a single-center study. An intact HPA axis and GC response are critical to the host response to infectious agents. Also, infectious agents may directly modulate GR functioning [36]. The importance of GRα functioning is clearly shown in septic mice lacking endothelial GRα demonstrating increased mortality, hemodynamic instability, pro-inflammatory cytokine production [37], and inability to downregulate IL-1β [18]. Our finding of increased levels of hGR, cortisol, extracellular HSPs, and cytokines in sepsis, especially among non-survivors, probably reflects the intensity of the activation of the HPA axis and acute stress inflammatory and innate-immune response. This increase might not have been attributed to the cell death, not only since the increased hGR levels were unrelated to LDH levels but also because the simultaneously measured abundant intracellular HSP72 and 90 proteins were repressed rather than increased in the sepsis group and among non-survivors.

GCs may induce apoptosis by directly regulating typical apoptosis or survival genes or by inducing cellular distress that triggers the apoptotic cascade [38]. Results of this study indicate that the high-risk patient prepares himself to respond to the “danger” by increasing the eHSP72, eHSP90α, and cortisol levels in a “ready to bind to an enhanced hGR” state. The higher eHSP72 and lower nHSP72 we found in patients receiving hydrocortisone might probably indicate a higher degree of stress and inflammation in this group. The positive relations of the hGR to the inflammatory biomarkers further support this, along with the simultaneous enhanced ACTH and prolactin, and the organ dysfunction or failure development, when the cell defending itself represses its bioenergetics. Results of this study, showing that cortisol and hGR are increased in the acute phase of sepsis, might indicate that cortisol is not “a limiting factor” early in sepsis. Thus, exogenous cortisol early in sepsis might repress the already enhanced hGR expression through a negative feedback mechanism, increasing the GC resistance. Although an increased hGR expression has also been shown in T lymphocytes during septic shock, regardless of GC treatment, hGR binding capacity was decreased in neutrophils in GC-treated patients [28]. We did not find any monocyte or whole blood hGR difference between patients started on stress hydrocortisone and those not receiving GCs. The decreased neutrophil capacity to bind hGRα in sepsis, however, might impede the response to GC treatment. Accordingly, the use of GCs in septic shock may be dependent on the stage of the sepsis, the reactivity of the HPA axis, and the sensitivity of hGR to the ligand [36]. Non-steroidal selective hGR modulators (Sin EGRAMs) that can activate specific hGR mechanisms and thus alter hGRα-mediated gene expression profiles have now gained more interest using mutant hGR research [39].

## Conclusions

In conclusion, early expression of hGRα mRNA or hGRα protein is significantly higher in patients with sepsis compared to healthy controls and/or patients with SIRS. Early in critical illness, the hGR expression can predict sepsis and outcome and is related with stress-activated bio-molecules and organ dysfunction. It is possible that the hGR, in association with the HSPs, is induced in monocytes to facilitate the cortisol binding to counter-balance the increased inflammatory response to the acute stress in early sepsis. Exogenous cortisol in the acute phase of sepsis might decrease the hGR expression through a negative feedback mechanism, increasing the GC resistance. Hopefully, smart research might in future lead to a more individualized approach to GC treatment in sepsis and septic shock, taking into account both the hGRα expression and binding capacity, as well as the balance of the immune-hormonal response.

### Publication of clinical datasets

Our dataset contains clinical data, for which we had undertaken an ethical and legal responsibility to respect participants’ rights to privacy and to protect their identity. The already received informed consents from participants at the point of recruitment to the trial did not mention any consent for publishing raw data. The institutional review board of Evangelismos Hospital had also demanded all data to be kept privately and not to be distributed. We should have known this possibility to include in our initial project and consent, which, however, we did not know. It is impossible now for the dataset to be considered for publication, because we may compromise anonymity or confidentiality or breach local data protection laws regarding patients’ and healthy individuals’ data. Patients and controls in an institution in a small period are well known, and it is now impossible to obtain a retrospective consent regarding publication of data.

## Abbreviations

AP-1: Activator protein-1
APACHE II: Acute Physiology and Chronic Evaluation
DBD: DNA-binding domain
GCs: Glucocorticoids
GRE: Glucocorticoid response elements
hGR: Human glucocorticoid receptor
HPA: Hypothalamic–pituitary–adrenal
HSP: Heat shock protein
ICU: Intensive care unit
ILs: Interleukins
LBD: Ligand-binding domain
LOS: Length of stay
NF-κB: Nuclear factor-κB
NLS: Nuclear localization sequences
RT-PCR: Real-time PCR
SAPS III: Simplified Acute Physiology Score III
SIRS: Systemic inflammatory response syndrome
SOFA: Sequential Organ Failure Assessment
STATs: Signal transducers and activators of transcription
